# Anti-NMDA receptor encephalitis: A case report

**DOI:** 10.1192/j.eurpsy.2021.1951

**Published:** 2021-08-13

**Authors:** H.L. Tan

**Affiliations:** National Addiction Management Service, Institute of mental heath, Singapore, Singapore

**Keywords:** encephalitis, anti-nmda receptor encephalitis

## Abstract

**Introduction:**

Anti-N-methyl-D-aspartate receptor (anti-NMDA-R) encephalitis is well-characterised autoimmune encephalitis with prominent psychiatric manifestations, neurological manifestations like speech dysfunction, seizures, dyskinesias and other movement abnormalities, decreased level of consciousness and autonomic instability. This disorder affects primarily children and adults up to 45 years. Females are 4 times more common than males and may have association with ovarian teratoma.

**Objectives:**

To identify anti-NMDA receptor encephalitis based on clinical features, facilitate early screening and relevant investigations to prevent delay in treatment.

**Methods:**

A case study of 36 year old female presented with clinical manifestations of autoimmune encephalitis syndrome.

**Results:**

Diagnosis confirmed by presence of NMDA receptor antibodies in serum and cerebrospinal fluid.

**Conclusions:**

Early recognition of clinical features of Anti-NMDA receptor encephalitis and early initiation of treatment has shown to improve outcomes, speed recovery and reduce the risk of relapses.

**Disclosure:**

No significant relationships.

